# Microglial exosomes: taking messaging to new spheres

**DOI:** 10.1093/braincomms/fcab041

**Published:** 2021-03-31

**Authors:** Deborah Kronenberg-Versteeg, Balazs Varga

**Affiliations:** 1 Hertie Institute for Clinical Brain Research, University of Tübingen, Tübingen, Germany; 2 German Center for Neurodegenerative Diseases, Tübingen, Germany; 3 Wellcome-MRC Cambridge Stem Cell Institute, University of Cambridge, Cambridge, UK

**This scientific commentary refers to ‘The influence of the R47H TREM2 variant on microglial exosome profiles’ by Mallach et al. (https://doi.org/10.1093/braincomms/fcab009)**.

The negative impact of neurodegenerative conditions on life quality has been increasing due to the lengthening of human lifespan. It is now well appreciated that both neurons and glia are fundamentally involved in neurodegenerative diseases. Microglia are essential for brain homeostasis through clearance of cellular debris, releasing neurotrophic factors as well as pruning of synapses. Studies have linked many of the genetic mutations that increase the risk for neurodegenerative diseases, such as Alzheimer’s disease, to a predominant expression of these genes by microglia. Variants in triggering receptor expressed on myeloid cell 2 (*TREM2*) gene are among the high-risk factors for late-onset Alzheimer’s disease.^1^ TREM2 is a transmembrane protein and important for cell survival, phagocytosis and proliferation. Polyanionic ligands, bacterial lipopolysaccharide (LPS) and phospholipids, as well as lipoproteins, including apolipoprotein E, low-density lipoprotein and amyloid-beta A4 protein, have been identified as endogenous ligands to TREM2.^1^ Ligand binding induces phosphorylation of the adaptor protein DAP12 and downstream signalling via several signalling cascades including activation of phosphatidylinositol 3-kinase and mitogen-activated protein kinases and the elevation of intracellular Ca^2+^, which regulate a multitude of cellular functions.^1^ The R47H variant is located in the protein’s extracellular immunoglobulin domain and thought to reduce the receptor’s binding affinity and is therefore considered a partial loss-of-function.^1^ Comparison of TREM2-deficient and R47H mutant rodent microglia to wild-type cells showed differential expression of several genes during homeostatic conditions. The effect of TREM2 deficiency increased after exposure of microglia to Alzheimer’s disease-associated mutated microtubule-associated protein tau (*MAPT*) or PSEN/APP forms indicating an altered behaviour of the mutant cells in pathological conditions.^1–3^

Comparison of murine and human microglia transcriptome revealed differences in gene expression^4^ and aberrant splicing between Exons 1 and 2,^5^ highlighting the importance to study microglia function directly in human cells. Derivation of neural and immune cells from induced pluripotent stem cells (iPSCs) allows the use of patient derived cells that carry genetic information to create tissue models for neurological conditions. The derivation of human iPSC-derived microglia-like cells has been designed to recapitulate microglial embryonic development. iPSC-derived microglia show several features of *in vivo* human microglia, including ramified morphology, expression of categorical microglial markers (such as IBA1, CX3CR1, CD11b, CD45, TMEM119) and typical microglial functions such as phagocytosis, process motility and cytokine response upon stimulation.

Besides physical contact, metabolic support and cytokine release, exosomes and extracellular vesicles (EVs) have recently emerged as an important mechanism for intercellular communication in the central nervous system.^6^ Microglial EVs have been implied in acute inflammatory responses through the release of IL-1β, regulation of neuronal synaptic activity,^7^ and as a source of energy substrates for neurons.^8^

In this issue, Mallach et al.^9^ present new data that focuses on the possible role of exosomes in neurodegenerative conditions. The authors took advantage of iPSCs established from patients carrying the TREM2 R47H mutation and differentiated the cells to microglia lineage.^10^ Previous analysis from the same workgroup showed reduced metabolic activity of microglia carrying the TREM2 mutation.^11^ In the current follow-up study the authors tested whether the TREM2 mutation can alter the content of microglia-released exosomes and if the exosomes have altered function. The TREM2 R47H mutant cells were found to have reduced exosome release and altered exosomal peptide content including nine proteins related to negative regulation of transcription and metabolism (Fig. 1). The altered exosome release could be rescued by supplying the cells with cyclocreatine, indicating that extracellular metabolic support could restore some TREM2 mutation-related microglial dysfunction.

**Figure 1:**
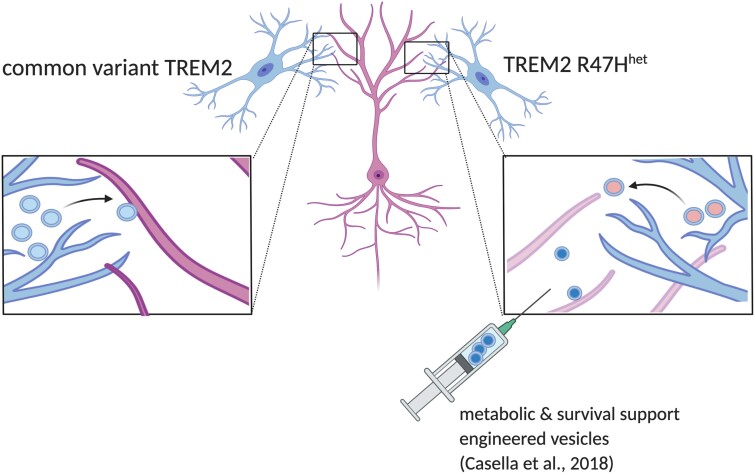
**Microglia release exosomes to support neurons.** Mallach et al. show differences in exosome content and neuronal support efficiency of TREM2 R47H mutant human microglia (figure made with www.biorender.com).

Microglia need to respond to various immunogenic stimuli in the brain to protect the function of neural cells. When microglia were activated with bacterial mimetic LPS, mutant cells responded by changing the content of their exosomes; however, the exosomes contained reduced quantity of chemokines (CCL22, CXCL5) and IL-1β cytokine compared to the common TREM2 variant indicating that the TREM2 mutation could alter the inflammatory response of microglia.

Microglia have been shown to have a protective role in the brain by supporting neurons with cytokines and by removing harmful biomaterial from around neurons; Mallach et al. (9) therefore investigated further if exosomes can support the survival of damaged neurons. To test if exosomes can transfer factors from microglia to stressed or apoptotic neurons, the authors tried to rescue apoptotic neuron-like cells with these exosomes. The R47H mutant showed a diminished rescue effect on neuronal survival and reduced MAP1A, TNFAIP8L2, TMPOa/b/g, UBQLN2 levels suggesting an altered immune function. Taken together with previous observations, these results indicate that microglia can release exosomes that carry material to support the survival of injured neurons, but that this function could be compromised in the R47H mutant and expose the neural cells to higher vulnerability after acute or chronic pathogenic stimuli (Fig. 1).

The study of Mallach et al. (9) highlights very relevant properties of human microglia-derived exosomes, which emphasize the necessity to investigate these further, but also point towards new therapeutic avenues involving the delivery of survival factors to injured neurons through membrane packaged vesicles, as has been tried recently in a mouse model for multiple sclerosis^12^ (Fig. 1).

Future studies will need to clarify the full metabolite, nucleic acid and proteome content of microglial exosomes to identify the factors mediating the trophic support or modulatory action of neural cell function. It will be interesting to test how the exosome content of mutant cells changes after stimulation by injured or apoptotic neurons and if the metabolic support can support TREM2 mutant microglia to rescue injured neurons. Furthermore, exosomes can be isolated from blood serum and cerebrospinal fluid, therefore, providing both an analytical and therapeutic angle in neurological conditions. Therapeutically, bioengineered exosomes present an exciting new approach to deliver chemicals or biologicals to injured neural cells by avoiding the sequestering or inhibition of metabolites and cytokines by surrounding cells or the extracellular matrix. This article and the potential therapeutic angle of it mark the beginning of a newly emerging field of extracellular vesicle research in neurological conditions.

## Data Availability

Data sharing is not applicable to this article as no new data were created or analysed.
